# Hip Pain in a Child Caused by Coxal Bone Infection

**DOI:** 10.5334/jbsr.2491

**Published:** 2021-09-17

**Authors:** Matthieu Wendling, Maxime Gudelj

**Affiliations:** 1 C.H.R. de la Citadelle, Liège, BE; 2 CHU de Liège, Liège, BE

**Keywords:** ischiopubic synchondrosis, osteomyelitis, magnetic resonance imaging

## Abstract

**Teaching Point**: Ischiopubic synchondrosis osteomyelitis is not rare but frequently misdiagnosed. MRI has a key role for early diagnosis and detection of complications.

## Case

A seven-year-old boy was admitted in our emergency department (ED) with right groin pain after sport without notion of trauma or fall. At clinical examination, the hip was painful in all movements. There was initially no fever, and the patient went back home with monitoring instructions without undergoing blood testing. Right hip radiographies and ultrasonography were negative. His past medical history was unremarkable.

The same day, the patient presented again at ED with fever (39.7°C) and significant increase in right groin pain.

**Figure 1:**
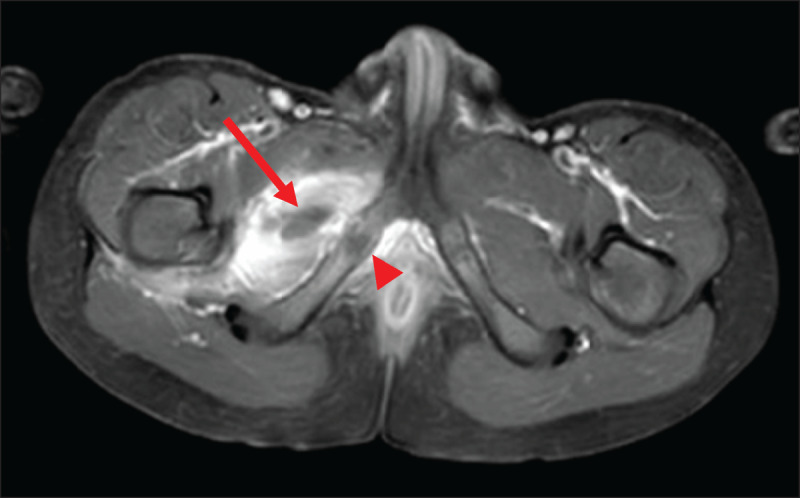


**Figure 2:**
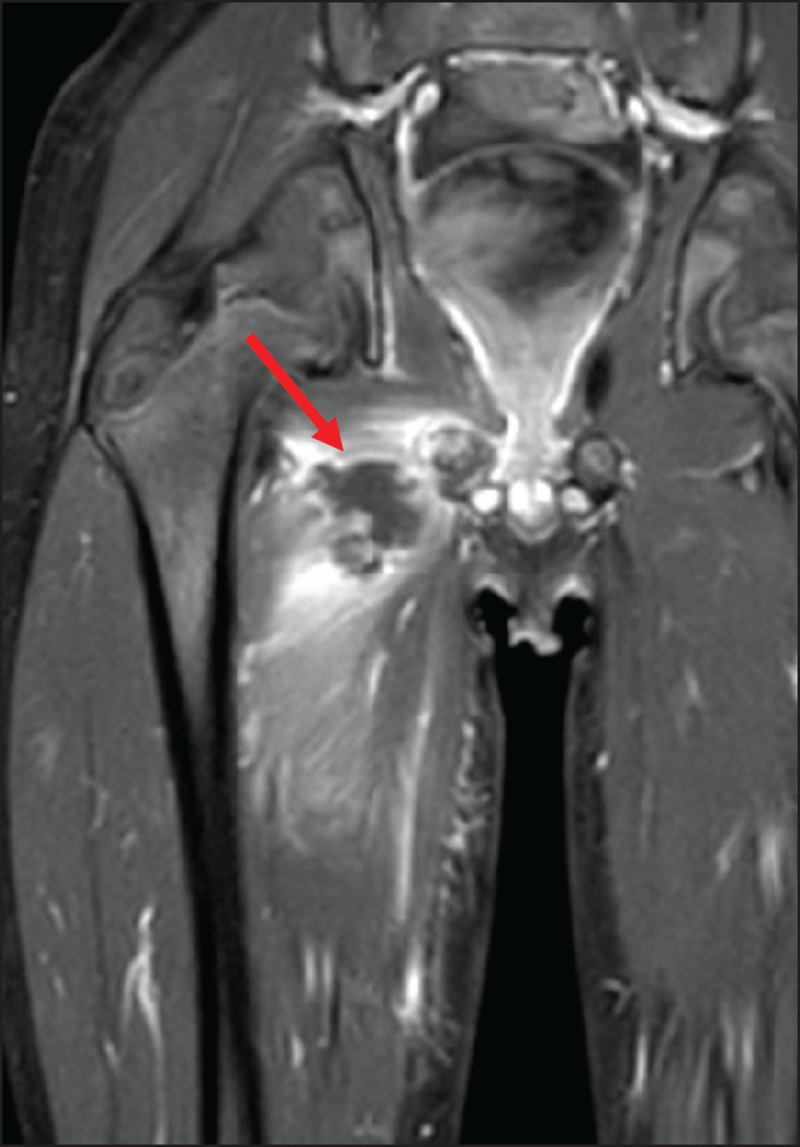


**Figure 3:**
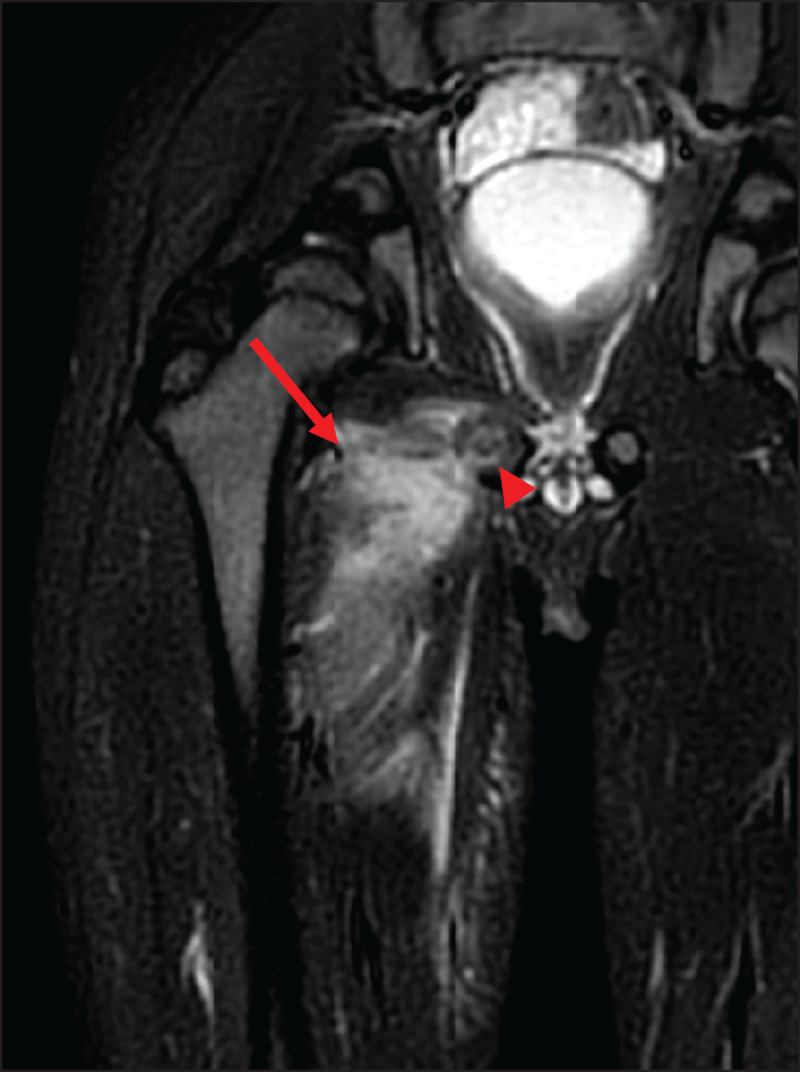


At this time, the blood analysis showed high white blood cells (18.500/mm3) and CRP (171.6 mg/L). Magnetic resonance imaging (MRI) (***Figures 1, 2, 3***) revealed a 3 cm long subperiosteal collection in contact with the right ischiopubic synchondrosis (arrowheads), suggestive of an abscess (arrows on axial. ***Figure 1***, and coronal, ***Figure 2***, post-contrast fat-saturated T1-weighted images). The abscess extended to the proximal part of adductors compartment and the right ischiopubic branch was involved by edema, appearing as high signal on short-tau inversion-recovery (STIR) imaging (***Figure 3***). Extensive edema was also visible in the surrounding tissues.

Blood culture revealed a staphylococcus aureus bacteriemia. An antibiotic treatment was administered, and the abscess was monitored with ultrasonography until disapearance, which was confirmed on follow-up MRI six weeks later.

## Comment

Ischiopubic synchondrosis (IPS) osteomyelitis is not very rare but the diagnosis is initially not obvious. The main differential diagnoses are hip infection and stress fracture (in an athletic patient).

Because of their rich vascular supply and relative blood stasis, metaphysis in long bones and metaphyseal equivalent regions in flat bones are susceptible for hematogeneous spread of infection in children [1].

The clinical presentation varies greatly and is not specific, comprising groin pain, abdominal pain, limitation of movement, fever, and swelling.

When present, radiographic signs are quite subtle, showing an unspecific and asymmetric enlargement of the IPS, which can be a simple variant of the normal.

Due to poor ability to study bone, ultrasonography has no real place in the initial diagnosis but eventually prove useful in the follow-up of possible intramuscular collections.

MRI is highly sensitive (82–100%) for early detection of osteomyelitis and its complications such as myositis or abscess. Osteomyelitis typically appears as an edematous high signal on T2-weighted and STIR sequences and on T1 post-gadolinium sequences of the synchrondrosis, with edema in surrounding tissues. When diagnosis and treatment are delayed, abscess can be seen (ring like pattern enhancement) in surrounding tissues. Bone erosions and new bone formation are sometimes also visible.
